# Supplementary material for: Changing patterns of general practice services during a period of public sector investment in Britain
Source: PLoS One. 2025 Jan 7;20(1):e0313168. doi: 10.1371/journal.pone.0313168 (PMC11706369; doi:10.1371/journal.pone.0313168)
Supplement: Attachment — Submitted filename: Response to Reviewers.docx [file pone.0313168.s001.docx]

**PLOS ONE**

**PONE-D-22-25682**

**Changing patterns of general practice services during a period of public sector investment in Britain**

**Reviewers' comments:**

Reviewer #1: This study uses data from the British Household Panel Survey to "examine GP and outpatient service use" and their relationship with different socio-demographic characteristics between 2000 and 2008, a period of organisational reform and increasing expenditure. I will explain two major reservations I have regarding this manuscript, and then make a number of more detailed comments.

**Reviewer comment:**

While it is true that the period 2000-2008 marked a time of greatly increased health expenditure on the UK NHS, it is not at all clear to me why this analysis should be restricted to this period. Surely also including the periods thereafter (e.g., 2012, 2016), a time of significant austerity (which the authors acknowledge in line 416-418) would provide even deeper insights on underlying relationships (or the lack thereof)? An investigation of putative changes in access related to spending investment would surely be informed equally by periods of low spending increase (or decreases) as by periods of fast growth? Given that this paper has been submitted in 2022, and several waves of BHPS data are available for intervening years, I find this choice hard to explain, and the authors provide no real discussion of why this decision was made.

**Response:**

We thank the reviewer for his comments and suggestions. We have updated our analysis to include data for 2015/16, 2016/17, and 2017/18. Our analysis is limited by changes to the way in which morbidity is recorded after Wave BH18 (2008) with the change over from British Household Panel Survey BHPS to Understanding Society Survey. This had informed our original decision to limit the scope of our analysis. We have, however now included details of the mean number of visits for GP and outpatients for the period before and after the adoption of austerity measures and included discussion of these results.

More details about the revision of this comment can be found below among other changes inserted to the manuscript.

I am also not really clear as to the fundamental purpose or research question driving this work. It is of course acceptable to undertake a descriptive analysis of relationships between variables, but I am left with the sense that the study was not guided by a clear underlying hypothesis or purpose. As a result, the results and conclusions of this manuscript seem somewhat disjointed and lack a coherent message. In their introduction, the authors cite official activity statistics to show large increases in GP activity in the NHS (but do not attempt to gather similar data for outpatient consultations, which are readily available for the NHS). They state in their discussion (lines 369-373): "our results demonstrate that in terms of the percentage of service users across the entire sample, utilization of GP and outpatient services increased only marginally" and "...only weak evidence that sustained investment and an expansion in service use increased access". They do not discuss the obvious corollary - which is that, while the number of people seeking consultations did not increase dramatically, the frequency of consultations increased amongst those who did (i.e. people who were ill received better access, while people who were not ill still did chose not to seek care). My interpretation of their results is that the % of people seeing a GP did increase; yet - surprisingly given that BHPS provides data on numbers of visits per respondent - they did not attempt to examine whether the frequency of visits per person increased. At the same time, their finding that the morbidity index fell is consistent with improving access - people who were less sick were more likely to have sought care in 2008 than 2000. Yet their Conclusion (lines 435-437) directly contradicts their own Discussion: "Our study demonstrated an increase in utilisation of GP and outpatient services...suggestive of widened access."

Unfortunately, I think this speaks to a limited degree of coherence and consistency underlying this paper. These major concerns unfortunately lead me to make a recommendation of "rejection" for this manuscript. A future revision of this manuscript would benefit not only from including additional years and an analysis of consultation frequency but would benefit from an improved attention to the context under discussion.

**Response**

We thank the reviewer for his comment, On reflection we agree that the aims of our paper required clarification and our conclusion needed to align more coherently with the discussion. We have therefore redrafted the introduction, method, result discussion, limitation, and conclusions as noted below:

**Introduction:** we have clarified the study hypothesis as quoted below:

“In this paper, we analyse three waves of the British Household Panel Survey (BHPS) and of its successor the understanding society survey to examine the relationship between GP and outpatient service use and a changing funding environment.”

**Method:**

“to compare utilisation in a period where resource constraints were more evident, we report mean number of GP and outpatient visits in 2000,2004,2008 and 2015/16, 2016/17, and 2017/18. Changes in questionnaire design from the British Household Panel Survey (BHPS) to its successor the Understanding Society Survey meant it is not possible to measure health in a consistent fashion across the two surveys. It was therefore not possible to repeat the multivariable analysis for earlier years for 2015 onward. However, the mean figures provide an indication of changes to use as the resource environment changed. Means were based on the class mark for categories that detailed the level of service use reported in the surveys.” “We use outpatient use to help understand GP use, where outpatient use affectively served as an indication of severity(10). We do not consider A&E use in this regard given services are not subject to gatekeeping by the GP.”

**Result:** In results, new tables have been added which report the mean utilisation for the two periods. And the following text: “Table 7 shows mean of General Practitioner (GP) visits and outpatient visits during periods characterized by relative resource abundance and resource scarcity. As can be seen, the mean number of GP visits fell by roughly 0.25 visits from (3.002) in 2000 to (2.757) in 2008. Conversely, regarding outpatient visits, the mean number increased by roughly 0.4 visits from (1.186) in 2000 to (1.229) in 2008.”

In the second period, while the average number of GP visits initially demonstrated an upward trend, ascending from (2.537) in 2015/16 to (2.698) in 2016/17, it declined dramatically to (1.195) in 2017/2018, a fall of more than one full visit. In contrast, the mean outpatient visits during the same period displayed slight growth, rising marginally by roughly 0.03 from (1.297) in 2015 to (1.333) in 2018.

**Discussion:**

“Comparing the two periods 2000 – 2008 and 2015 -2018 shows mean GP utilisation fell more dramatically in second period while outpatient use remained relatively static – perhaps reflecting the ability of GPs to re-direct demand for services elsewhere in the system.

The decline in demand-led GP use in the second period and the virtually unchanged level of outpatient use suggests that a more constrained resource environment may have been an important factor in influencing service use across the two periods.

“while the GP utilisation fell slightly during the period of relative resource abundance the fall in use was more dramatic during the period of resource constraint. Outpatient utilisation increased but not significantly during the period of relative resource abundance and a similar pattern was observed for the period of resource constraint. This is supportive of the argument that during the period of relative abundance utilisation of GP services was able to increase.”

While it was not possible to model service use in the same way across the two time periods due to the manner in which health was measured in BHPS compared to Understanding Society, patterns are evident in respect of socio-demographic characteristics in the earlier period.”

“It is unfortunate that the data available did not allow us to model use consistently across periods of distinct relative resource scarcity.”

“where as shown the mean number of visits fell sharply over a relatively short period of time.”

“As GP services have come under further strain in the wake of the COVID pandemic such differences may have been experienced to an even greater extent.”

**Limitations:**

it is not possible to model service use in a consistent fashion using the BHPS successor the Understanding Society Survey. Unfortunately, this transition coincides with a period of austerity where resource constraints became more evident.”

**Conclusion:**

“Our study demonstrated that use of GP services is related to distinct periods of relative resource abundance and resource scarcity. In the period of resource abundance, that those with specific conditions including hearing and vision impairment were consistently less likely to use outpatient services suggests the possibility of ongoing unmet need in respect of certain conditions. As resource scarcity has increased with respect to GP services in particular following the COVID pandemic suggests such patterns of use warrant close examination.”

**Specific comments:**

Line 116-127 - the discussion of QoF etc. is somewhat limited and one-sided: an explicit aim of QoF (better quality chronic disease management) could explicitly be framed (and was at the time) as a means of reducing referrals to secondary (outpatient) care by means of better primary care management. This possibility is not considered.

**Response:**

Thank you for drawing our attention to this, we have now extended the discussion to link our finding with implication related to QoF as the following:

“While our analysis links the resource environment to patterns of utilization it is important to also remember the broader context in which services were provided and consumed. Over the period studied, for example, the population increased and aged, with rising multimorbidity contributing to increased needs and the complexity of those needs. Similarly, broader policy changes such as the introduction of the Quality Outcomes Framework changed the incentive structure under which GPs operated, providing financial rewards for the identification and management of chronic conditions such as diabetes.  These could each affect patterns of service use and complicate the establishment of causal relationships as does the fact that the data we use does not provide the opportunity for the consistent modelling of utilization. These limitations should be born in mind when reflecting on our findings.”

Line 161-166 - this section suggests a misunderstanding which appears throughout the paper (e.g. also lies 391-2) Access to outpatient care is not entirely dependent on access to a GP in the NHS. Most critically, attendance at Accident & Emergency can lead directly to referral to outpatient services, as can a range of other smaller services 9e.g. family planning, STI clinics etc.)

**Response to reviewer:**

We thank the reviewer for drawing our attention to this oversight the following text. “We use outpatient use to help understand GP use, where outpatient use effectively serves as an indication of severity. We do not consider A&E use in this regard given services are not subject to gatekeeping by the GP though it is acknowledged that this may provide a route by which some service users may also be referred to outpatient services.”

Table 1: How can the "morbidity index squared" be 1.73 when the morbidity index value is 0.82? The square of 0,82 is 0.67

***Response to reviewer:***

We thank the reviewer for drawing our attention to this mistake, it has been corrected.

Table 4: I found the presentation of hours of work and Age confusing - these appear to have been split into quintiles, but - especially for age - this seems unusual, and no explanation is given of what age range fits each age quintile.

**Response to reviewer:**

Thank you for your comment, we have added information for the classification of two variables: hours of working and age.

- For hours of working: Q1 - 0-22 hours; Q2: 23-35 hours; Q3: 36-37 hours; Q4: 38-40 hours; Q5: 41+ hours.
- For age group: Group (1) 15-28 years old, group (2) 29-40 years old, group (3) 41-51 years old, group (4)52-65, and group (5) 66+ years old,

We used quintiles to account for the possibility of non-linear associations. We hypothesized that as hours of work or age increased, the likelihood of visiting a healthcare provider might exhibit a different rate of decline or increase. By utilizing quintiles, we aimed to capture these potential variations and provide a more nuanced understanding of the relationship. The use of quintiles allows for the consideration of factors such as the impact of time constraints on healthcare-seeking behaviour and the accumulation of chronic conditions with age. By grouping the data into quintiles, we can identify patterns and trends across distinct sub-groups, enabling a comprehensive analysis of the morbidity index.
