# Supplementary material for: Changing patterns of general practice services during a period of public sector investment in Britain
Source: PLoS One. 2025 Jan 7;20(1):e0313168. doi: 10.1371/journal.pone.0313168 (PMC11706369; doi:10.1371/journal.pone.0313168)
Supplement: Attachment — Submitted filename: Response to Reviewers.docx [file pone.0313168.s002.docx]

**PLOS ONE**

**PONE-D-22-25682**

**Changing patterns of general practice services during a period of public sector investment in Britain**

***Reviewers' comments:***

Reviewer #1: Thank you for the efforts that the authors have gone to in addressing my original comments. The paper is greatly improved by the addition of later data, notwithstanding the limitations of the new version of the survey. I would strongly encourage the authors to spend a little more time on their Discussion and Conclusions sections however - I find these a little thin and not really bringing out all of the key issues. What does it mean that outpatient utilisation consistently increased while GP utilisation fell over time? It means that relative accessibility of primary care is decreasing, and use of hospital-based ambulatory care is increasing. This is a critical finding - because it shows that reality has been the exact opposite of decades of policy efforts seeking to decrease secondary care use by strengthening primary care. I would like to see these deeper policy implications discussed rather more directly. It was also not clear to me whether the authors are making their data available online or in a supplement - this should be addressed before acceptance.

***Response:***

Thank you for your valuable feedback and for acknowledging the improvements made to the paper. We appreciate your suggestion to further strengthen the Discussion and Conclusions sections and have added additional text to these.

Regarding the availability of used data. Data from the British Household Panel Survey were collected by the Institute for Social and Economic Research at the University of Essex and made available by the UK Data Archive. We have added a note that the data can be located from this source.
