# Supplementary material for: Changing patterns of general practice services during a period of public sector investment in Britain
Source: PLoS One. 2025 Jan 7;20(1):e0313168. doi: 10.1371/journal.pone.0313168 (PMC11706369; doi:10.1371/journal.pone.0313168)
Supplement: Attachment — Submitted filename: Response to Reviewer.docx [file pone.0313168.s003.docx]

PONE-D-22-25682R2

Changing patterns of general practice services during a period of public sector investment in Britain

PLOS ONE

**Review Comments to the Author**

Reviewer #2: This study presents a compelling analysis of the shifting patterns in service utilization correlating with variations in investment levels. However, the lack of a standardized population for comparison undermines the strength of these findings.

Additionally, the rationale for selecting data from the years 2015, 2016, and 2017 remains unexplained. It would enhance the study's relevance if the authors could present data on annual spending for primary care services rather than relying on broader economic indicators like GDP healthcare expenditure.

Furthermore, this information is accessible in the NHS's annual reports. To improve the study's comprehensiveness and accuracy, the authors are encouraged to include:

The number of general practitioners available during these specified years.
The population size for each of these years.

**Response to reviewer comment:**

**Reviewer comment:**

This study presents a compelling analysis of the shifting patterns in service utilization correlating with variations in investment levels. However, the lack of a standardized population for comparison undermines the strength of these findings. Additionally, the rationale for selecting data from the years 2015, 2016, and 2017 remains unexplained.

**Response:**

We thank the reviewer for his comments. Our analysis is limited by changes to the way in which morbidity is recorded after Wave BH18 (2008) with the change over from British Household Panel Survey BHPS to Understanding Society Survey. This had informed our original decision to limit the scope of our analysis. We have, however included details of the mean number of visits for GP and outpatients for the period before and after the adoption of austerity measures and included discussion of these results.

This explanation is included in the manuscript in lines 219–229 of the discussion section:

“To compare utilisation in a period where resource constraints were more evident we report mean number of GP and outpatient visits in 2000,2004,2008 and 2015/16, 2016/17, and 2017/18. Changes in questionnaire design from the British Household Panel Survey (BHPS) to its successor the “Understanding Society Survey” meant it is not possible to measure health in a consistent fashion across the two surveys. It was therefore not possible to repeat the multivariable analysis for earlier years for 2015 onward. However, the mean figures provide an indication of changes to use as the resource environment changed. Means were based on the class mark for categories that detailed the level of service use reported in the surveys. We use outpatient use to help understand GP use, where outpatient use affectively served as an indication of severity (10). We do not consider A&E use in this regard given services are not subject to gatekeeping by the GP”.

In lines 424–428 of the discussion section:

“While it was not possible to model service use in the same way across the two time periods due to the manner in which health was measured in BHPS compared to Understanding Society, patterns are evident in respect of socio-demographic characteristics in the earlier period. Further research could extend our analysis to examine the impact on use of accident and emergency services.”

Additionally, this point is reiterated in lines 497–500:

“It is not possible to model service use in a consistent fashion using the BHPS successor the Understanding Society Survey. Unfortunately, this transition coincides with a period of austerity where resource constraints became more evident.”

Additional text has been added in the method section:

“We selected the years 2015, 2016, and 2017 because they represent a period during which austerity measures introduced in the early 2010s had accumulated and impacted service utilization. These years provide a clearer understanding of how service use evolved under the influence of constrained public spending. Additionally, taking three consecutive years minimizes the risk of arbitrary selection, as it allows us to observe any emerging trends rather than focusing on a single year that might not be representative of the broader context”.

**Reviewer comment:**

To improve the study's comprehensiveness and accuracy, the authors are encouraged to include: The number of general practitioners available during these specified years.
The population size for each of these years.

**Reponse:**

We have added the requested information in the results section. The additional text reads:

“Data on the availability of General Practitioners (GPs) during this period indicate a relatively stable workforce. In 2015, there were 34,492 GPs (FTE), which increased slightly to 34,916 GPs (FTE) in 2017. This suggests that the observed decline in GP visits in 2017/18 is unlikely to be solely attributed to changes in GP availability”.
